# HCG18 Participates in Vascular Invasion of Hepatocellular Carcinoma by Regulating Macrophages and Tumor Stem Cells

**DOI:** 10.3389/fcell.2021.707073

**Published:** 2021-08-30

**Authors:** Liwei Zhang, Zhiwei Wang, Mingxing Li, Peng Sun, Tao Bai, Wang Wang, Hualong Bai, Jianjun Gou, Zhiju Wang

**Affiliations:** ^1^Department of Vascular and Endovascular Surgery, The First Affiliated Hospital of Zhengzhou University, Zhengzhou, China; ^2^Key Vascular Physiology and Applied Research Laboratory of Zhengzhou City, Zhengzhou, China; ^3^Department of Physiology, Medical School of Zhengzhou University, Zhengzhou, China; ^4^Department of Clinical Laboratory, The First Affiliated Hospital of Zhengzhou University, Zhengzhou, China

**Keywords:** HCG18, vascular invasion, HCC, macrophages, immune infiltration, tumor stem cells

## Abstract

**Objectives:**

To identify key genes involved in vascular invasion in hepatocellular carcinoma (HCC), to describe their regulatory mechanisms, and to explore the immune microenvironment of HCC.

**Methodology:**

In this study, the genome, transcriptome, and immune microenvironment of HCC were assessed by using multi-platform data from The Cancer Genome Atlas (*n* = 373) and GEO data (GSE149614). The key regulatory networks, transcription factors and core genes related to vascular invasion and prognosis were explored based on the CE mechanism. Survival analysis and gene set enrichment were used to explore pathways related to vascular invasion. Combined with single-cell transcriptome data, the distribution of core gene expression in various cells was observed. Cellular communication analysis was used to identify key cells associated with vascular invasion. Pseudo-temporal locus analysis was used to explore the regulation of core genes in key cell phenotypes. The influence of core genes on current immune checkpoint therapy was evaluated and correlations with tumor stem cell scores were explored.

**Results:**

We obtained a network containing 1,249 pairs of CE regulatory relationships, including 579 differential proteins, 28 non-coding RNAs, and 37 miRNAs. Three key transcription factors, ILF2, YBX1, and HMGA1, were identified, all regulated by HCG18 lncRNA. ScRNAseq showed that HCG18 co-localized with macrophages and stem cells. CIBERSORTx assessed 22 types of immune cells in HCC and found that HCG18 was positively correlated with M0 macrophages, while being negatively correlated with M1 and M2 macrophages, monocytes, and dendritic cells. Cluster analysis based on patient prognosis suggested that regulating phenotypic transformation of macrophages could be an effective intervention for treating HCC. At the same time, higher expression of HCG18, HMGA1, ILF2, and YBX1 was associated with a higher stem cell score and less tumor differentiation. Pan cancer analysis indicated that high expression of HCG18 implies high sensitivity to immune checkpoint therapy.

**Conclusion:**

HCG18 participates in vascular invasion of HCC by regulating macrophages and tumor stem cells through three key transcription factors, YBX1, ILF2, and HMGA1.

## Introduction

Hepatocellular carcinoma (HCC) is a highly aggressive malignant tumor with high mortality (Akinyemiju et al., 2017). The prognosis of advanced HCC is poor, with a 5-year survival rate of about 15%. Current comprehensive treatment, which mainly focuses on surgery supplemented by chemoradiotherapy and immunotherapy, has improved survival time. Unfortunately, postoperative recurrence is common, with a recurrence rate as high as 50% 5 years after surgery, and postoperative transplantation is required in 10–20% of cases (Dhir et al., 2016). Therefore, increased understanding of the mechanisms leading to progression and recurrence of hepatocellular carcinoma (HCC) would be helpful for developing new molecular markers and therapeutic targets to improve patient survival.

Vascular invasion, present in 25–50% of HCCs (Hsu et al., 2013), divided into macrovascular and microvascular invasion. Macrovascular invasion refers to the tumor thrombus formed by the portal vein and its main branches. Microscopically, the presence of nests of cancer cells in vascular lumens lined with endothelial cells is defined as microvascular invasion. Plasma biomarkers currently used clinically such as alpha-fetoprotein (AFP) and decarboxyloprothrombin (DCP), have low sensitivity and are insufficient for determination of the aggressiveness of HCC (Kim et al., 2017). Imaging diagnosis can improve the diagnostic accuracy of tumor aggressiveness (Banerjee et al., 2015), but tumor heterogeneity leads to great differences between individuals. Therefore, identification of new molecular markers related to tumor invasion and prognosis would be helpful for accurate diagnosis and personalized treatment of cancer patients, as well as provide for new therapeutic directions.

Previous studies have assessed clinical risk factors and genetic characteristics of HCC vascular invasion (Wang et al., 2007; Yuan et al., 2012; Han et al., 2019). Although the molecular mechanisms of HCC progression has been extensively studied, the specific genes involved in vascular invasion are still unclear. Studies of the tumor immune microenvironment have shown that immune cell infiltration is related to vascular invasion. However, in HCC, the role of key genes related to vascular invasion through regulation of immune-infiltrating cells has not been reported.

In this study, we comprehensively analyzed the genomic and transcriptome characteristics of HCC as well as the tumor immune microenvironment. A regulatory network based on a CE mechanism has been established. Combined with transcription factor analysis, three key proteins, YBX1, ILF2, and HMGA1, involved in vascular invasion were identified, and gene set enrichment analysis (GSEA) of their regulatory networks showed that all three were regulated by HCG18. When HCG18 expression was high, activation of the vascular endothelial growth factor (VEGF) pathway increased. Single cell sequencing showed that macrophages and tumor stem cells are key to vascular invasion. Macrophage subtype analysis showed that expression of HCG18 and vascular endothelial growth factor A (VEGFA) was positively correlated with infiltration of M0 macrophages and negatively correlated with M1 and M2 macrophages, monocytes and dendritic cells (DCs). Cluster analysis of immune infiltration into HCC tumors showed that promoting transformation of M0 to M2 macrophages could inhibit vascular invasion and improve prognosis. The tumor stem cell score based on the one-class logistic regression (OCLR) algorithm showed that HCG18 and its regulated transcription factors HMGA1, ILF2, and YBX1 positively correlated with the degree of tumor undifferentiation. Pan cancer analysis indicated that high expression of HCG18 implies high sensitivity to immune checkpoint therapy.

The results of our study suggest that HCG18 may be a new target for intervening in vascular invasion and improving prognosis of liver cancer.

## Materials and Methods

### Data Acquisition

Transcriptome data (RNAseq, miRNAseq) was downloaded from The Cancer Genome Atlas (TCGA) database^1^, and the clinical data of the corresponding samples was obtained from the CBioPortal website^2^. The single-cell sequencing data GSE149614 was downloaded from the GEO database. The data used to build the CE regulatory network were downloaded from several websites: miRcode, lncBase^3^, TargetScan^4^, miRand^5^, miRDB, and miRTarBase^6^. Transcription factor analysis used the KnockTF website^7^. Immune cell gene expression files required by CIBERSORTx software were downloaded from the CIBERSORT website^8^. The TIDE score was calculated at http://tide.dfci.harvard.edu. Gepia 2.0^9^ was used for expression correlation and survival analysis. Additional quantitative analysis utilized R and related packages, Cytoscape, and GSEA software.

### Clinical Analysis of Vascular Invasion in Hepatocellular Carcinoma

Clinical information for the samples was analyzed by multivariate Cox survival analysis. Both micro- and macro-vascular invasion were included in the vascular infiltration group. For analysis of the effect of vascular infiltration on overall survival time and disease-free progression of patients with hepatocellular carcinoma, we used R and the R package Survminer, Survival.

### Construction of the CE Regulatory Network and Analysis of Transcription Factors

MicroRNAs are short RNAs with lengths of about 22 nucleotides that can regulate the expression of target genes by competing with mRNA for the same microRNA response elements (MREs). In order to construct the CE regulatory network, the regulatory relationships of miRNA and its targets (miRNA-mRNA or miRNA-lncRNA) were firstly predicted. Then the lncRNA-mRNA regulatory relationships were predicted according to the shared miRNA. At the same time, correlations between the regulatory relationships were calculated to screen the results and improve reliability of the prediction.

We filtered the CE-pairs according to the following criteria:

(A) LncRNA-miRNA interactions were predicted by miRcode and LncBase (experimental support), mRNAs targeted by miRNAs were predicted by miRDB (score >75), TargetScan (context percentile >50), and miRanda (energy <−15). The expression of two types of interactions should be negatively correlated.
(B) LncRNA-mRNA interactions had to satisfy the following conditions:
  (a) At least one miRNA was shared.
  (b) Expression of LncRNA and mRNA were positively correlated (correlation ≥0 and *P* < 0.05).
  (c) A hypergeometric test was performed to assess whether a LncRNA and mRNA share miRNAs (*P* < 0.05).
  (d) Regulation similarity analysis was performed to check the similarity between expression of miRNAs-LncRNA and miRNAs-mRNA (correlation ≥0.5).

Patients were grouped according to vascular invasion, and differential expression of mRNA (*P* < 0.05), LncRNA (*P* < 0.05), and miRNA (*P* < 0.05) was obtained. To identify core proteins, survival analysis of proteins in the regulatory network was performed (K-M, *P* < 0.05) (Supplementary Tables 1–3). Transcription factor analysis was conducted by KnockTF Analysis. This process was supported by R.

### Clinical, Genomic and Transcriptome Characteristics of HCG18

HCG18 is considered to be a key regulator of vascular invasion. The clinical significance of HCG18 was demonstrated by analysis of overall survival and disease-free progression. The CBioPortal was used to explore the genomic characteristics of HCG18 in HCC, including single nucleotide mutations and copy number changes, and to rank patients according to whether they had vascular invasion. In addition, GSEA was used to evaluate the effect of HCG18 overexpression on the transcriptome. Gene sequencing between the groups was generated by the signal to noise algorithm. KEGG (Kyoto Encyclopedia of Genes and Genomes) databases were considered to have significant differences when GSEA *P* < 0.05 (Supplementary Table 4).

### Single-Cell Transcriptome Analysis of HCC

The GSE149614 dataset was originally designed for exploring the relationship between microenvironment and tumorigenesis, including the primary tumor, portal vein thrombosis, metastatic lymph node and normal control, with a total of 21 samples from 71,915 cells. Ten tumor samples with a total of 31,491 cells were included in this study. The gene filter condition was satisfied in at least 500 cells, the cell filter condition was satisfied in at least 500 genes, and not more than 10% of transcripts were of mitochondrial origin. After data standardization, dimension reduction was conducted by the UMAP algorithm and clustering. SingleR was used to annotate cells based on the tag genes in each cluster. A violin diagram was used to analyze the distribution of expression levels of HCG18, HMGA1, ILF2, and YBX1 in each subtype of cells.

### Cell Communication Analysis

To identify key cells involved in vascular invasion, intercellular communication analysis was performed based on single-cell transcriptome data. CellChat^10^ uses cell gene expression data as input and simulates cell-to-cell communication by combining the interactions of ligand receptors and their cofactors. After the intercellular regulatory relationships were obtained, the key pathways involved in vascular invasion were screened and the key cells communicating with the endothelium were identified. By combining with the expression distribution of HCG18, HMGA1, ILF2, and YBX1, we explored the cell types regulated by these genes in the process of vascular invasion. Pseudo-temporal trajectory analysis was used to further explore the regulatory mechanism of HCG18, HMGA1, ILF2, and YBX1 at the cellular level.

### Analysis of the Tumor Immune Microenvironment Based on CIBERSORTx

By analyzing the expression distribution and cell communication of HCG18, HMGA1, YBX1, and ILF2, we concluded that these genes are involved in vascular invasion by regulating the function of mononuclear macrophages and tumor stem cells. To further determine the specific types of cells involved in vascular invasion, we used CIBERSORTx to evaluate the infiltration rate of 22 types of immune cells, including M0, M1, and M2 type macrophages, into the tumor. The relationship between HCG18 and VEGFA expression and the amount of infiltration of various types of macrophages suggested that promoting differentiation of macrophages to the M2 type may be a possible approach to intervening in vascular invasion of HCC and improving prognosis. This process was supported by R and the R package Seurat, Monocle CellChat, and CIBERSORT algorithms.

### Tumor Stem Cell Scoring Based on the OCLR Algorithm

Stem cells have the potential to self-renew and differentiate. Cancer progression involves the gradual loss of differentiated phenotypes and the acquisition of progenitor cell-like and stem-cell-like characteristics. Undifferentiated primary tumors are more likely to cause cancer cells to spread to distant organs, leading to disease progression and poor prognosis. Our study shows that tumor stem cells are involved in vascular invasion in HCC. HCG18, HMGA1, ILF2, and YBX1 were expressed in tumor pluripotent stem cells, and cell communication analysis showed that stem cells were key to vascular invasion. In this study, we used a machine learning OCLR algorithm to calculate the tumor stem cell score for each TCGA-HCC sample by establishing a prediction model for pluripotent stem cell samples (ESC and IPSC) from the PCBC dataset. The relationship between HCG18, HMGA1, ILF2, YBX1, and VEGFA expression and tumor stem cell score was verified at the bulk level.

### Correlation Analysis Between HCG18 and Immune Checkpoint Therapy

To investigate the effect of HCG18 on immunotherapy, we performed a pan cancer analysis. HCG18 – associated tumors were screened by overall and progression-free survival analyses based on TCGA. The correlation between HCG18 and CD274, PDCD1, CTLA4, MSI, and TMB were analyzed in these tumors. The TIDE algorithm uses a comprehensive analysis of hundreds of different tumor profiles to find biomarkers that can predict the efficacy of immune checkpoint inhibitors, determining whether T cells are failing in immune-hot tumors and whether three types of cells are inhibiting T cell infiltration in immune-cold tumors (Jiang et al., 2018). RNAseq data was analyzed using the TIDE website^11^ to obtain the TIDE rating of each sample. Higher TIDE predictive scores were associated not only with poorer immune checkpoint suppression, but also with poorer survival in patients treated with anti-PD1 and anti-CTLA4.

## Results

### Vascular Invasion of Hepatocellular Carcinoma Has Independent Prognostic Significance

**FIGURE 1:**
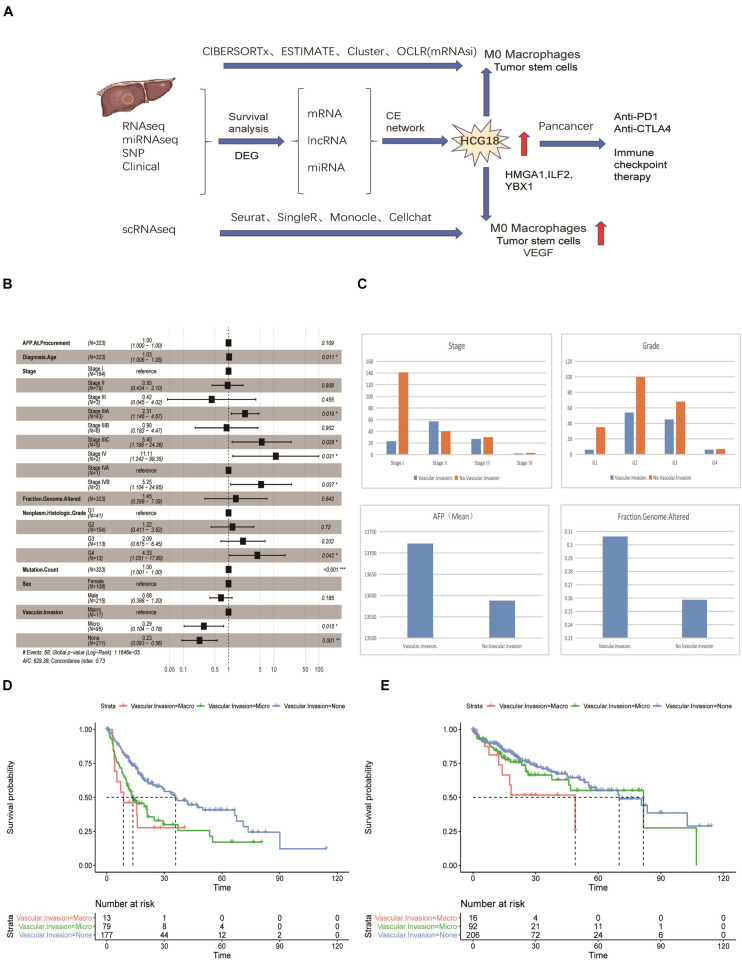
Vascular invasion has independent prognostic significance in hepatocellular carcinoma. **(A)** Data flow chart for this study, including data types, algorithms, and part of the R package. **(B)** Forest map based on multi-factor Cox regression of clinical characteristics of TCGA samples. Compared with the reference value, relative risk less than 1 was considered to be protective, while relative risk greater than 1 was considered to be a risk factor. *P* < 0.05 was considered statistically significant. **(C)** Histogram of stratified frequency statistics of vascular invasion in staging and grading, histogram of vascular invasion, AFP and genomic changes. **(D)** Disease-free survival analysis based on vascular invasion stratification. **(E)** Overall survival analysis based on stratification of vascular invasion. *P < 0.05; **P < 0.01; ***P < 0.001.

Previous studies have confirmed that vascular invasion of HCC tumors is associated with recurrence and shorter survival. However, the relationship between vascular invasion and other clinical features has not been fully studied. To this end, we performed a multivariate Cox survival analysis of clinical information recorded in samples from the TCGA database (Figure 1A). We found that vascular invasion had independent prognostic significance relative to stage and grade (*P* < 0.05) (Figure 1B). Analysis of the frequency of vascular invasion of samples at different stages and grades showed that the incidence of vascular invasion increased with an increase in pathological grade. At the same time, AFP was higher in the invasion group, as was genomic instability (Figure 1C). By analyzing recurrence and overall survival times, it was shown that micro-vessel invasion did not affect overall survival time compared with the non-vascular invasion group, but the disease-free survival time was shorter and the recurrence time was earlier. Recurrence time and overall survival were significantly reduced in patients in which macrovascular invasion had occurred (Figure 1D). In general, both macro- and micro-vessel invasion had independent prognostic significance, and macro-vessel invasion had the poorest prognosis (Figure 1E).

### HCG18 Is a Key Gene for Vascular Invasion in Hepatocellular Carcinoma

**FIGURE 2:**
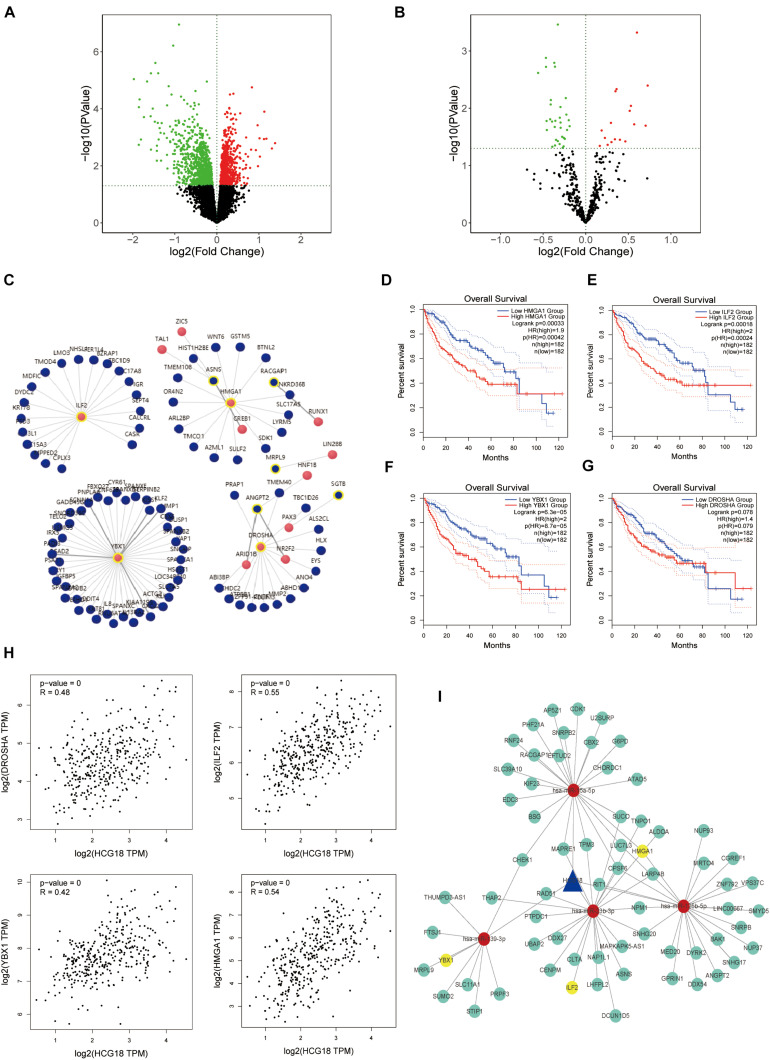
HCG18 is the core lncRNA for vascular invasion in hepatocellular carcinoma. **(A)** Volcanic map of transcriptome differential expression based on vascular infiltration (RNASeq); *P* < 0.05 was the standard for screening genes. Green represents low expression of vascular versus non-vascular invasion genes, and red represents high expression. **(B)** Volcanic map of differentially expressed transcriptomes based on vascular invasion (miRNASeq). **(C)** Analysis of transcription factors where red represents transcription factors and yellow circles represent those involved in the core CE regulatory network. **(D)** Overall survival analysis of HMGA1. **(E)** Overall survival analysis diagram of ILF2. **(F)** Overall survival analysis diagram of YBX1. **(G)** Overall survival analysis diagram of DROSHA. **(H)** Scatter plot of HCG18 and expression levels of YBX1, ILF2, HMGA1, and DROSHA. **(I)** The core regulatory network involving transcription factors (YBX1, ILF2, and HMGA1) that regulate vascular invasion. Yellow represents transcription factors, red represents miRNA, and blue represents lncRNA. All three transcription factors are regulated by HCG18.

In this study, we explored the key genes involved in vascular invasion by constructing a CE regulatory network. The transcriptome data of the vascular invasion group was compared with that of the non-invasion group, and *P* < 0.05 was used for screening of mRNA, lncRNA, and miRNA (Figures 2A,B). The analysis yielded 1,715 protein-coding genes, 82 non-coding genes, and 50 miRNAs (Supplementary Figure 1). LncRNA, target miRNAs, and miRNA target proteins were predicted using the micode, lncBase, TargetScan, miRAND, miRDB, and miRTARbase databases, and the intersection among these was used to construct CE regulatory relationship pairs. The final result was 10,301 pairs of regulatory relationships. Screening conditions included LncRNA and mRNA expression (COR > 0, *P* < 0.05), hypergeometric distribution (*P* < 0.05), and regulation similarity coefficient (COR > 0.5 *P* < 0.05), which left 1,249 pairs of regulation relationships. Finally, survival analysis of the proteins in the network was carried out to select the regulatory network that incorporated mRNA with survival significance. This process retained 393 pairs of regulatory relationships considered to be key to vascular infiltration of HCC. In order to further explore the core genes, this study focused on transcription factors (Figure 2C). After transcription factor analysis, three key transcription factors, YBX1, ILF2, and HMGA1 (Figures 2D–G), were obtained and found to be regulated by hsa-miR-139-3p, hsa-miR-23b-3p, and hsa-miR-125b-5p, respectively. These transcription factors are all regulated by the same HCG18 lncRNA (Figures 2H,I).

### HCG18 Affects the Transcriptome Characteristics of Hepatocellular Carcinoma

**FIGURE 3:**
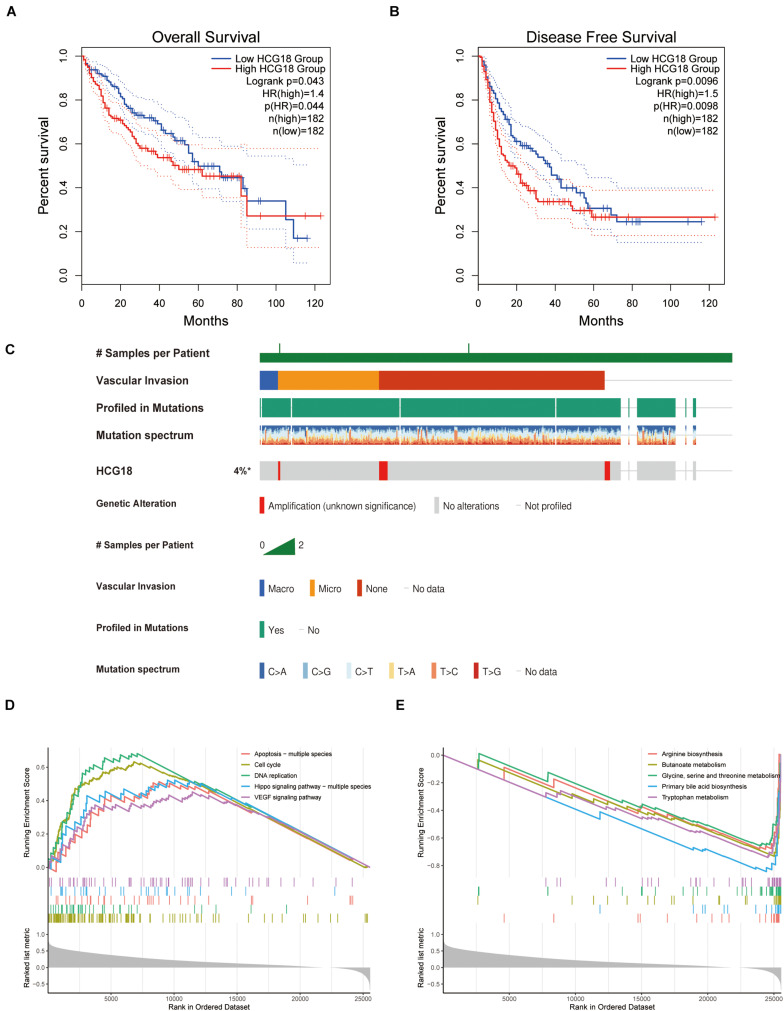
Clinical, genomic and transcriptome characteristics of HCG18. **(A)** Overall survival analysis of HCG18. **(B)** Disease-free progression-free survival analysis of HCG18. **(C)** Single nucleotide mutations and copy number changes of HCG18 in HCC patients, ranked by vascular invasion. **(D)** GSEA analysis based on KEGG; an enrichment score greater than 0 represents a pathway that increases expression of HCG18 after high expression. **(E)** An enrichment score less than 0 represents a pathway of decreased HCG18 expression after high HCG18 expression. In GSEA analysis, *P* < 0.05 was used as the criterion to screen for significantly altered pathways. *The amplification of HCG18 in HCC was statistically significant.

HCG18 is a key regulatory gene for vascular invasion in hepatocellular carcinoma, and high expression of HCG18 was found to have independent prognostic significance (Figures 3A,B). To explore the mechanism of HCG18 overexpression, we investigated the genomic characteristics of HCG18 in tumor samples. HCG18 mutations and copy number changes were mapped by the multi-omics analysis website CBioPortal, and patients were ranked according to whether or not they had vascular invasion. As shown in Figure 3, HCG18 showed no significant genomic changes in HCC patients and no defining genomic characteristics during vascular invasion (Figure 3C). High expression of HCG18 was not associated with genomic variation. Further GSEA analysis revealed that high HCG18 expression significantly affected transcriptome characteristics, including cell cycle, DNA damage repair, high expression of RNA-stable pathways, and increased expression of VEGF and TGFβ pathways (Figure 3D). In addition, it is worth noting that high expression of HCG18 lead to reduced amino acid metabolism and expression of bile synthesis pathways, suggesting that HCG18 may be a marker for metabolomics subtype classification of hepatocellular carcinoma, and that it plays a key regulatory role in maintaining liver function (Figure 3E).

### HCG18 Regulates VEGF Expression by Macrophages and Tumor Stem Cells

By analyzing the GSE149614 single-cell sequencing dataset, 31,491 cells from 10 samples were obtained. After standardization and UMAP (Uniform Manifold Approximation and Projection) dimensionality reduction and clustering, 31 clusters of cells were obtained. The classification core genes were extracted, and 7,078 tag genes were obtained. The cell types were annotated by the SingleR package. The heat map showed that the main cell types were liver cancer cells (14,041), monocytes (1,537), macrophages (5,550), T cells (3,972), and NK cells (881) (Supplementary Figure 2).

Expression of HCG18, HMGA1, ILF2, and YBX1 in all kinds of cells was analyzed (Supplementary Figure 3). Expression of HCG18 was low in tumors overall, but relatively high in the mononuclear macrophage system, T cells, NK cells, and tumor stem cells. HMGA1 and ILF2 were highly expressed in tumor stem cell-like cells, while YBX1 was highly expressed in both monocyte macrophages and tumor stem cell-like cells.

**FIGURE 4:**
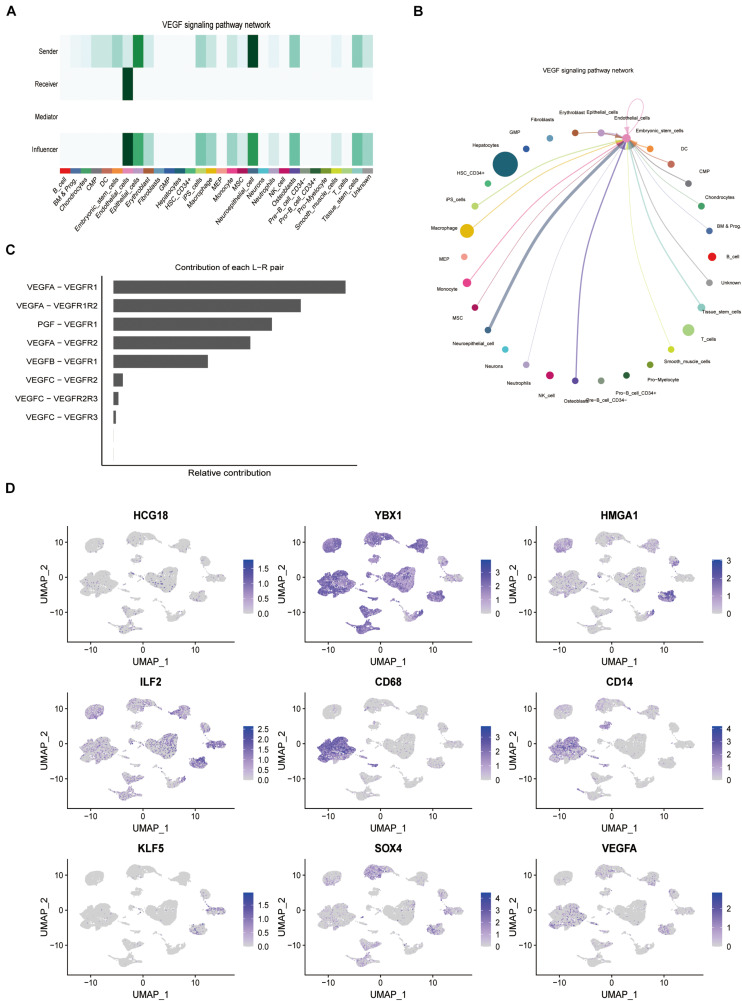
HCG18 can regulate the expression of VEGF in tumors by mononuclear macrophages and stem cells in tumors. **(A)** The role of cells in the VEGF pathway; darker colors represent higher confidence intervals. **(B)** Intercellular communication in the VEGF pathway. Thicker lines between two cells represent greater intercellular communication. **(C)** Contributions of ligand and receptor pairs to intercellular communication in the VEGF pathway. Cell communication in the VEGF pathway is mainly mediated by VEGFA-VEGFR1. **(D)** Combined analysis of expression distribution and intercellular communication showed that HCG18, HMGA1, ILF2, and YBX1 were mainly mediated by mononuclear macrophages and tumor stem cell-like cells in vascular invasion. CD68 and CD14 were used as molecular markers of macrophages and monocytes, respectively. KLF5 and SOX4 were used as stem cell-like markers.

Forty-six pathways related to cell interactions were identified by cell communication analysis. Among them, vascular invasion is related to the pro-angiogenesis VEGF pathway and the anti-angiogenesis VEGI (vascular endothelial growth inhibitor) pathway. Mononuclear macrophages and tumor stem cell-like cells in the VEGF pathway are the core cells (Figures 4A–C), while cells related to the VEGI pathway are not related to HCG18 and its regulation of transcription factor expression. In this study, CD68 and CD14 were used as markers for macrophages and monocytes, and KLF5 and SOX4 were used as markers for stem cell-like cells. HCG18 and VEGFA were observed to co-localize with these molecular markers (Figure 4D). These results suggest that HCG18 is involved in vascular invasion by regulating monocyte macrophages and stem cell-like cells in hepatocellular carcinoma.

### HCG18 Is Associated With M0 Macrophages

**FIGURE 5:**
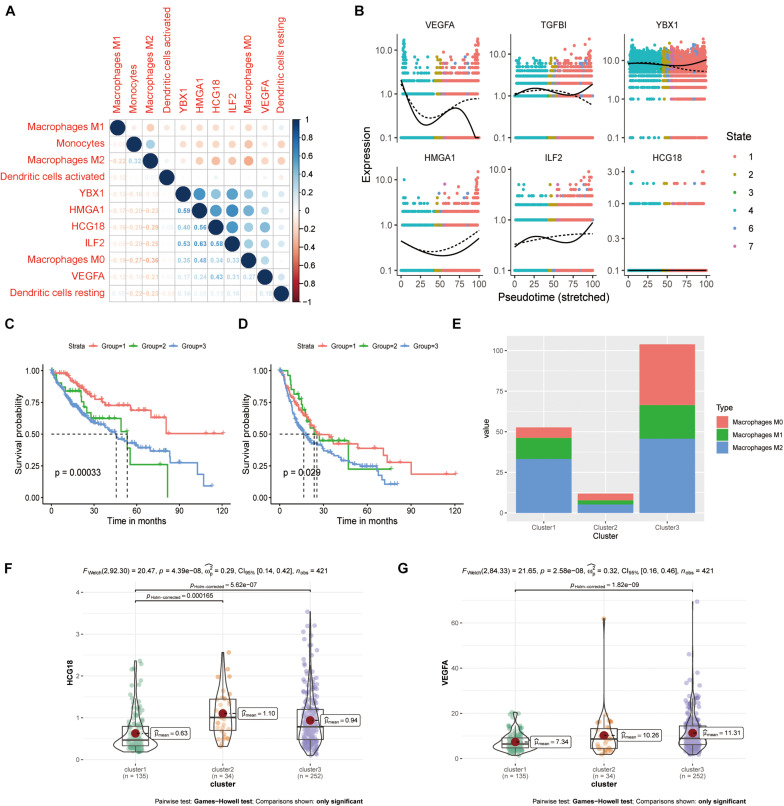
HCG18 is associated with M0 macrophages in the mononuclear macrophage system. **(A)** Correlation analysis of the expression of HCG18, HMGA1, ILF2, YBX1, and VEGFA upon infiltration of monocyte macrophages and DCs in tumors. **(B)** Pseudo-time locus analysis of HCG18, HMGA1, ILF2, and YBX1 regulating phenotypic differentiation of monocytes and macrophages. **(C)** Cluster analysis based on immune infiltrating cells divided the patients into three subtypes. Overall survival curves of the three subtypes are shown. **(D)** Survival curves of the three subtypes in disease-free progression. **(E)** Cumulative histogram of infiltrating macrophages in the three subtypes. **(F)** HCG18 expression level in the three subtypes. **(G)** Expression of VEGFA in the three subtypes.

We evaluated the infiltration score of 22 immune cells in TCGA samples and analyzed their correlation with expression of HCG18 and the transcription factors HMGA1, ILF2, and YBX1 (Supplementary Figure 4). The results showed that expression of these genes was positively correlated with M0 macrophages, negatively correlated with M1 and M2 macrophages and monocytes, and had no clear correlation with DCs (Figure 5A). Pseudo-time trajectory analysis showed that expression of HCG18 and its regulatory transcription factors varied with the stage of differentiation of monocyte macrophages, and that HCG18 could regulate the differentiation trajectory of monocyte macrophages (Figure 5B). Cluster analysis based on immune-infiltrating cells divided the patients into three subtypes (Supplementary Table 5) having significantly different prognosis (Supplementary Figure 5 and Figures 5C,D). The proportions of M0, M1, and M2 macrophages were analyzed (Figure 5E), and it was found that in Cluster1 patients, the proportion of M2 macrophages was higher, expression of VEGFA and HCG18 was lower, and the prognosis was relatively good. In Cluster3 patients, the proportion of M0 macrophages was higher, the expression of VEGFA and HCG18 was higher, and the prognosis was poor (Figures 5F,G). Our study suggests that stimulating an increase in M2 macrophages in tumors may contribute to inhibiting vascular invasion of HCC and to improving prognosis.

### HCG18, HMGA1, ILF2, and YBX1 Were Positively Correlated With Tumor Stem Cell Scores

**FIGURE 6:**
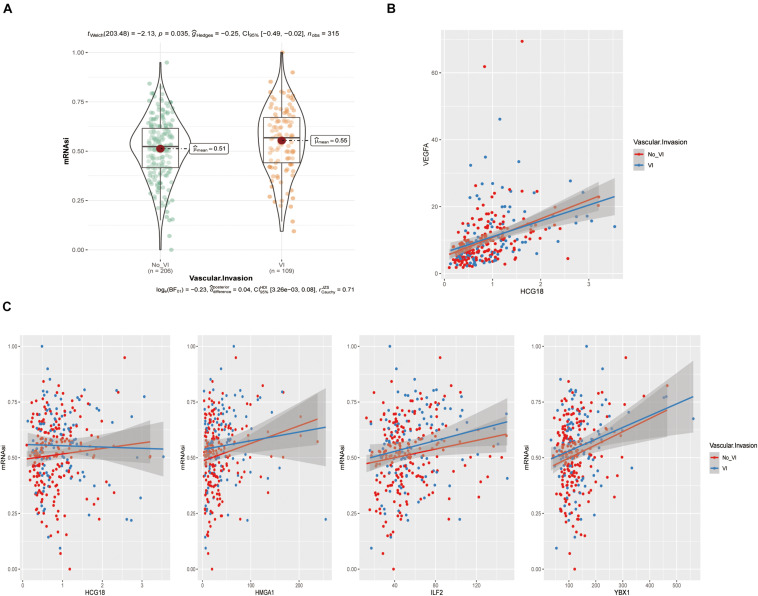
HCG18, HMGA1, ILF2, and YBX1 were positively correlated with tumor stem cell score produced by the OCLR algorithm. **(A)** Violin plot of stem cell scores in the vascular and non-vascular invasion groups. **(B)** Scatter diagram of HCG18 and VEGFA expression levels. **(C)** Scatter diagram of HCG18, HMGA1, ILF2, and YBX1 expression levels and stem cell scores. The regression line indicates a positive correlation.

The above analysis showed that HCG18, HMGA1, ILF2, and YBX1 could participate in vascular invasion via tumor stem cells. We evaluated stem cell scores in TCGA patients using the OCLR algorithm (Figure 6A and Supplementary Table 6) and performed correlation analysis of gene expression (Figures 6B,C). It was found that HMGA1, ILF2, and YBX1 were positively correlated with the score, and higher expression was associated with less differentiated tumors. However, there was no significant correlation between HCG18 expression and stem cell score (correlation coefficient 0.06, *P* = 0.12), which may be related to the low basal expression level of HCG18.

### High Expression of HCG18 Implies High Sensitivity to Immune Checkpoint Therapy

**FIGURE 7:**
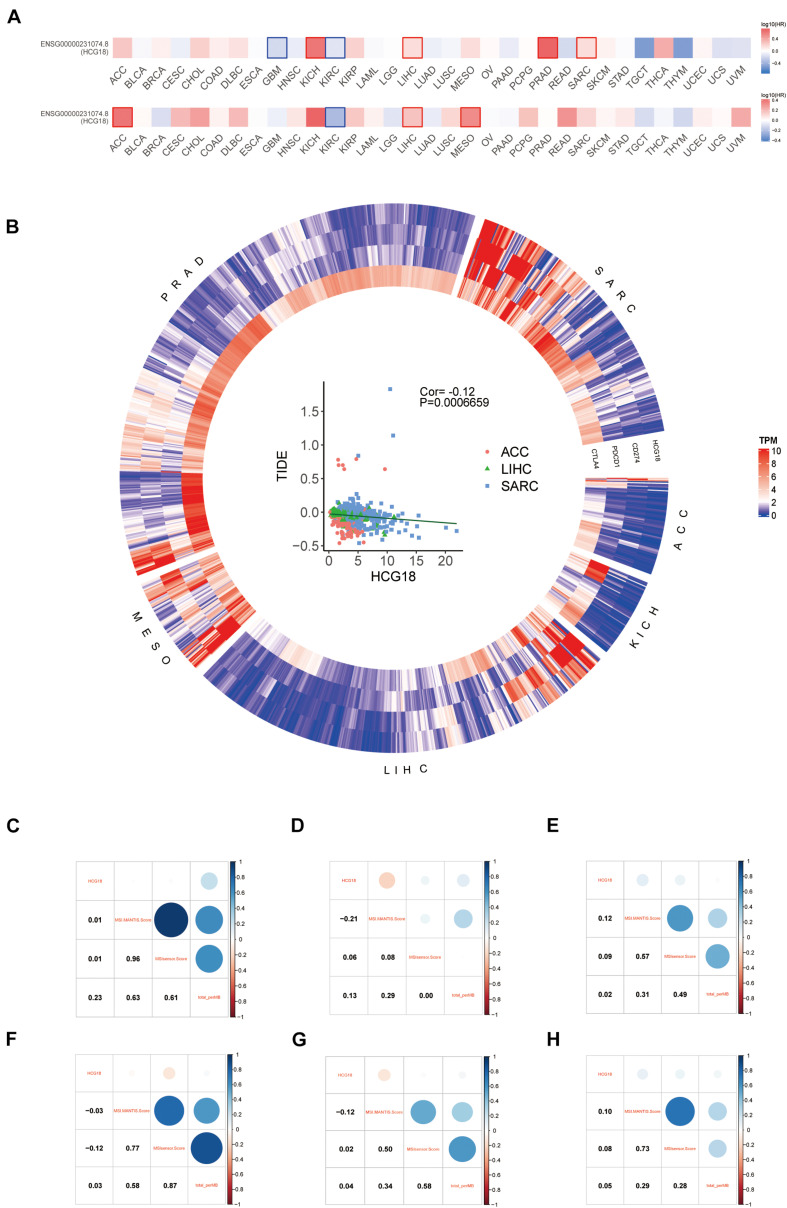
Pan cancer analysis of the effect of HCG18 on immunotherapy. **(A)** Survival map of overall and progression-free survival of HCG18 based on TCGA. **(B)** Heat maps of HCG18, CD274, PDCD1, and CTLA4 expression in six HCG18-related cancers (*P* value < 0.05). Scatter plot of TIDE score and HCG18 expression in LIHC, ACC, and SARC (COR = –0.12, *P* value < 0.001). **(C–H)** The correlation graph of HCG18 expression with MSI score and TMB in ACC, KICH, LIHC, MESO, PRAD, and SARC, respectively.

The sensitivity of tumors to immune checkpoint therapy is known to be associated with PDCD1, CD274, CTLA4, MSI, and TMB. To investigate the effect of HCG18 on immune checkpoint therapy, we performed a pan cancer analysis based on TCGA. Six HCG18-related tumors were screened by overall and progression-free survival analysis (ACC, KICH, LIHC, MESO, PRAD, and SARC, *P* value < 0.05) (Figure 7A). HCG18 was positively correlated with the expression of CD274, PDCD1, and CTLA4. TIDE score was negatively correlated with HCG18 expression (Figure 7B and Supplementary Table 7). As shown in Figures 7C–H, HCG18 was positively correlated with MSI score in LIHC and SARC, and positively correlated with TMB in ACC and KICH. In conclusion, the high expression of HCG18 indicates the increased sensitivity of tumor to immune checkpoint therapy.

## Discussion

Hepatocellular carcinoma (HCC) is one of the most common complex diseases in the context of chronic liver disease. Over the past 20 years, our understanding of the clinical and molecular heterogeneity of HCC has improved dramatically. The most commonly used clinical classification scheme is the Barcelona Clinical Liver Cancer (BCLC) system (Forner et al., 2012; Reig et al., 2014), which determines the stage of a cancer based on tumor load, the severity of the liver disease, and the patient’s clinical presentation. BCLC 0 or BCLC A have little vessel invasion or extrahepatic spread and retain liver function, and these patients can benefit from treatment (excision, transplantation, or ablation) (Bruix et al., 2016). Patients with BCLC B are asymptomatic, but the tumor is large, multifocal, and has no vascular invasion or extra-hepatic spread. If liver function is preserved, BCLC B patients can benefit from transarterial chemoembolization (TACE) (Dufour et al., 2013; Labgaa et al., 2019). Patients with BCLC C have tumors that have spread to the liver and/or invaded the vasculature, and tyrosine kinase inhibitors can prolong survival. Patients with BCLC D have poor liver function, poor prognosis and need supportive treatment. Although vascular invasion is not used as a primary reference criterion, our study shows that vascular invasion has independent prognostic significance relative to TNM (tumor, node, and metastasis) stage and pathological grade. Currently, Sorafenib, which is widely used in clinical practice, can inhibit the Raf/MEK/ERK-mediated cell pathway to directly inhibit tumor proliferation, and can also bind VEGF and PDGF receptors to inhibit abnormal angiogenesis in tumors. In addition, apatinib (Xu et al., 2021), an antagonist of the VEGF receptor (VEGFR), and bevacizumab (Casak et al., 2021; Castet et al., 2021), a VEGF/VEGFR monoclonal antibody, have achieved significant clinical results. It has been suggested that reducing abnormal vascular hyperplasia and invasion might be an effective direction for treatment of HCC, however, the mechanisms of abnormal angiogenesis and vascular invasion remain largely unstudied.

In this study, we mined three key transcription factors, YBX1, ILF2, and HMGA1, by constructing a vascular invasion-related CE regulatory network that included transcription factors.

YBX1 encodes a highly conserved cold-shock domain protein that binds to a wide range of nucleic acids, including both DNA and RNA, and mainly acts as an RNA binding protein to recognize mRNA transcripts modified by C5-methylcytosine (M5C) (Ray et al., 2009). mRNA stability is improved by recruiting the mRNA stability maintenance factor ELAVL1 (Chen et al., 2019, 2000), which is a component of a CRD-mediated complex that promotes MYC mRNA stability (Weidensdorfer et al., 2009). Recent genomic and proteomic studies of HCC have identified MYC as a key protein involved in vascular invasion that plays a critical role in gene-edited mice (Krishnan et al., 2020). In addition, YBX1 also regulates selective splicing of pre-mRNA by controlling interactions between mRNA and eukaryotic initiation factors (Raffetseder et al., 2003). It also plays a role in DNA repair (Gaudreault et al., 2004). The protein encoded by ILF2 is a transcription factor required by T cells to transcribe the interleukin-2 gene. Studies have shown that interference with ILF2 or ILF3 significantly reduces the expression of IL-6 and VEGF in tumors (Guarnerio et al., 2016). HMGA1 is a chromatin regulator that plays a role in several biological processes, including tumorigenesis, inflammation, and metabolism (Pang et al., 2012; Guarnerio et al., 2016; Zanin et al., 2019). Studies have shown that HMGA1 can enhance hypoxia-induced HIF-1-mediated VEGF expression (Messineo et al., 2016). The single nucleotide mutation rs139876191 in HMGA1 can down-regulate expression of VEGFA and inhibit proliferative diabetic retinopathy (Chiefari et al., 2016).

Three transcription factors, YBX1, ILF2, and HMGA1, have all been shown to be involved in vascular dysplasia, and our study also shows that they play a key role in vascular invasion. The CE regulatory network revealed that YBX1, ILF2, and HMGA1 were all regulated by HCG18. Therefore, HCG18 is believed to play a key regulatory role in vascular invasion. RNASeq and scRNASeq showed that overall expression of HCG18 was not high in tumors, but was specifically distributed in mononuclear macrophages and tumor stem cells, which were identified as being key to vascular invasion based on cellular communication analysis. Correlation analysis of HCG18 expression at the bulk level and immune infiltration showed that HCG18 expression was positively correlated with M0 macrophage infiltration, although negatively correlated with infiltration of M1 and M2 macrophages and monocytes. Cluster analysis based on immune infiltration showed that the proportions of M0, M1, and M2 macrophages affected prognosis. When the proportion of M0-type macrophages increased and the proportion of M2 macrophages decreased, the expression of VEGFA increased and prognosis was poor. The pseudo-time trajectory suggested that HCG18 and its regulated transcription factors can regulate the phenotypic transformation of macrophages.

In recent years it was found that the immune microenvironment in tumors plays an important role in regulation of angiogenesis. Angiogenesis is mainly related to the secretion of VEGF, TGFβ and other cytokines by immune cells that infiltrate tumors, and tumor-associated macrophages play a key role in this process (Ostuni et al., 2015; Mantovani et al., 2017). Based on phenotype and function, macrophages are usually classified into M1 and M2 types. IFN-γ, LPS, TNF-α, or GM-CSF promote differentiation of M1 type macrophages, which can promote an inflammatory response by secreting TNF-α, IL-1α, IL-1β, IL-6, IL-12, IL-18, IL-23, and other cytokines. IL-4, IL-13, M-CSF/CSF1, IL-10, IL-33, IL-21, TGF-β, and other cytokines can induce M2-type macrophages. M2 macrophages secrete IL-10, PGE2, and TGF-β, which are mainly involved in tissue remodeling and regeneration, wound healing and anti-inflammatory responses (Lawrence and Natoli, 2011; Mills et al., 2016). Recent studies have shown that under certain conditions, these different phenotypes may transform into one other.

Tumor-associated macrophages (TAMS) are usually the major component of myeloid cells in tumors. Macrophages have been shown to be directly or indirectly involved in several key features of malignancy, including angiogenesis, invasiveness, metastasis, regulation of the tumor microenvironment, and therapeutic resistance (Akalu et al., 2017; Keeley et al., 2019). This makes TAMS a potential target for cancer therapy. Macrophages participate in angiogenesis by expressing Wnt7b, Wnt5a, Wnt11, VEGF-C, VEGF-D, and other factors (Ran and Montgomery, 2012). Our study has shown that expression of VEGF in tumors is positively correlated with macrophage infiltration, which was consistent with the previous conclusion.

Immunotherapy is considered to be the most promising cancer treatment. A variety of cancer immunotherapies, including adoptive cell immunotherapy, tumor vaccines, antibodies, immune checkpoint inhibitors, and small molecule inhibitors, have had some success (Wang and Wang, 2017; Hegde and Chen, 2020; Kennedy and Salama, 2020). Therapeutic strategies based on macrophages or in combination with macrophages have the potential to improve the efficacy of cancer therapy (Feng et al., 2019). Recent reviews of targets and drugs related to the treatment of macrophage tumors have been published (Ngambenjawong et al., 2017). Our results suggest that promoting M2 phenotypic transformation of macrophages may contribute to inhibition of vascular invasion in tumors and to improved prognosis. HCG18 and its regulated transcription factors can regulate phenotypic conversion in the mononuclear macrophage system. In addition, these genes have been implicated in the stem cell signature of tumors.

Our study shows that high expression of HCG18 implies high sensitivity to immune checkpoint therapy. Compared with the currently known CD274, PDCD1, CTLA4, MSI, and TMB, HCG18 is not a direct target of immunotherapy, and as a non-coding RNA, its basic expression level is low. However, HCG18, as a non-coding RNA, increases the evaluation dimension of immunotherapy. Meanwhile, our study showed that HCG18 can be used as an evaluation indicator for vascular invasion, while other currently known immune evaluation indicators have no clear relationship with vascular invasion. In conclusion, HCG18 can simultaneously evaluate tumor angiogenesis, invasion and immune microenvironment.

The results of our study suggest that HCG18 should be a new target for treatment of hepatocellular carcinoma.

### Limitations

The conclusions obtained in this study are based on bioinformatics analysis. The protein expression level of the gene, its corresponding regulatory relationship and its influence on cell phenotypic transformation need to be further explored experimentally.

## Data Availability Statement

The datasets presented in this study can be found in online repositories. The names of the repository/repositories and accession number(s) can be found in the article/Supplementary Material.

## Author Contributions

LZ, HB, JG, and ZJW: conceptualization. LZ: methodology, software, formal analysis, and original draft preparation. ZWW, ML, and PS: validation. TB: investigation and resources. WW: data curation. JG and ZWW: review and editing. LZ and PS: visualization. ML: supervision. HB: project administration and funding acquisition. All authors contributed to the article and approved the submitted version.

## Conflict of Interest

The authors declare that the research was conducted in the absence of any commercial or financial relationships that could be construed as a potential conflict of interest.

## Publisher’s Note

All claims expressed in this article are solely those of the authors and do not necessarily represent those of their affiliated organizations, or those of the publisher, the editors and the reviewers. Any product that may be evaluated in this article, or claim that may be made by its manufacturer, is not guaranteed or endorsed by the publisher.

## Footnotes

1
https://portal.gdc.cancer.gov

2
www.cbioportal.org

3
http://carolina.imis.athena-innovation.gr/

4
http://www.targetscan.org/

5
http://cbio.mskcc.org/

6
http://mirtarbase.cuhk.edu.cn/

7
http://www.licpathway.net/

8
https://cibersortx.stanford.edu/

9
http://gepia2.cancer-pku.cn/

10
https://github.com/sqjin/CellChat

11
http://tide.dfci.harvard.edu
