# Supplementary figures and images for: HCG18 Participates in Vascular Invasion of Hepatocellular Carcinoma by Regulating Macrophages and Tumor Stem Cells
Source: Front Cell Dev Biol. 2021 Aug 30;9:707073. doi: 10.3389/fcell.2021.707073 (PMC8435853; doi:10.3389/fcell.2021.707073)

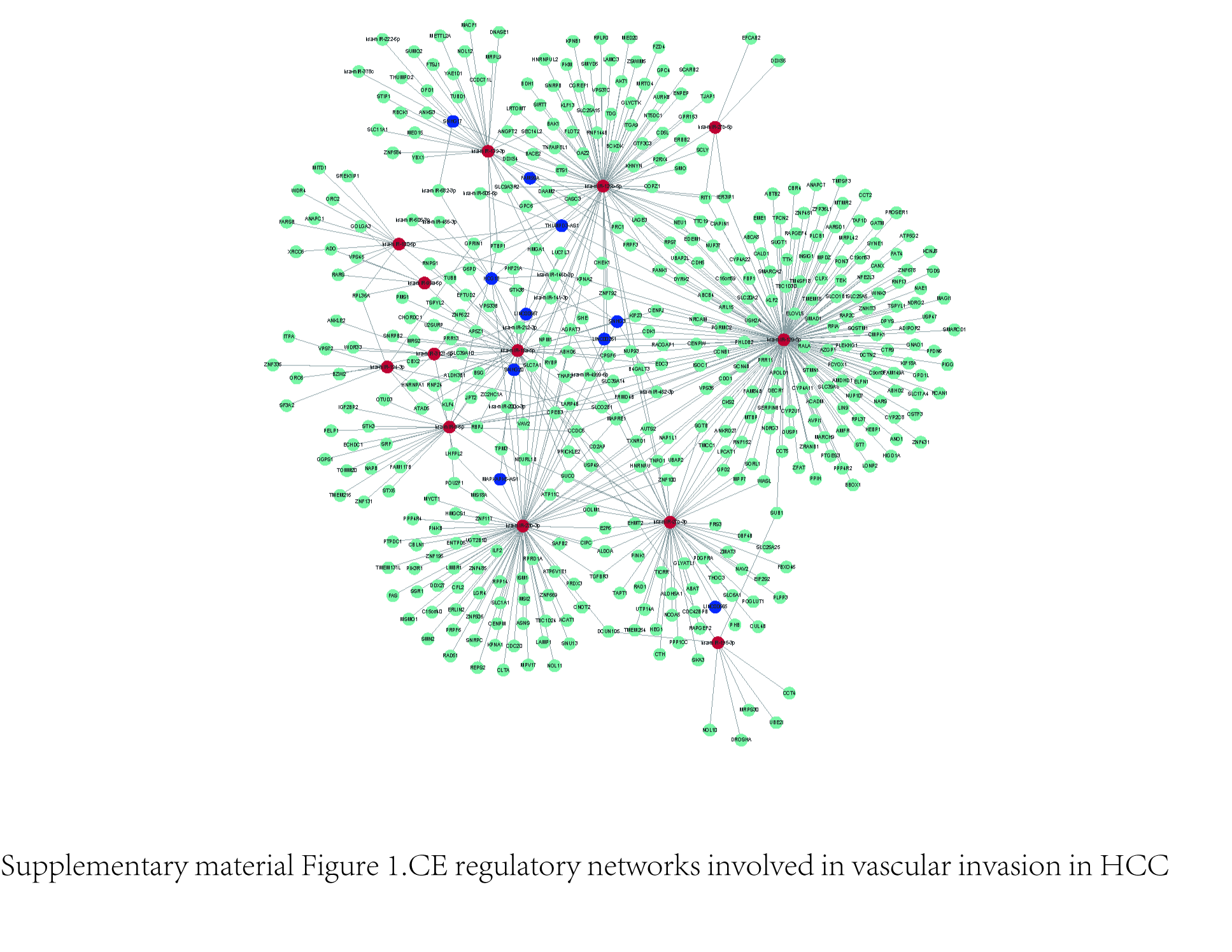

Supplement: Supplementary file 8 [file Image_1.TIF]

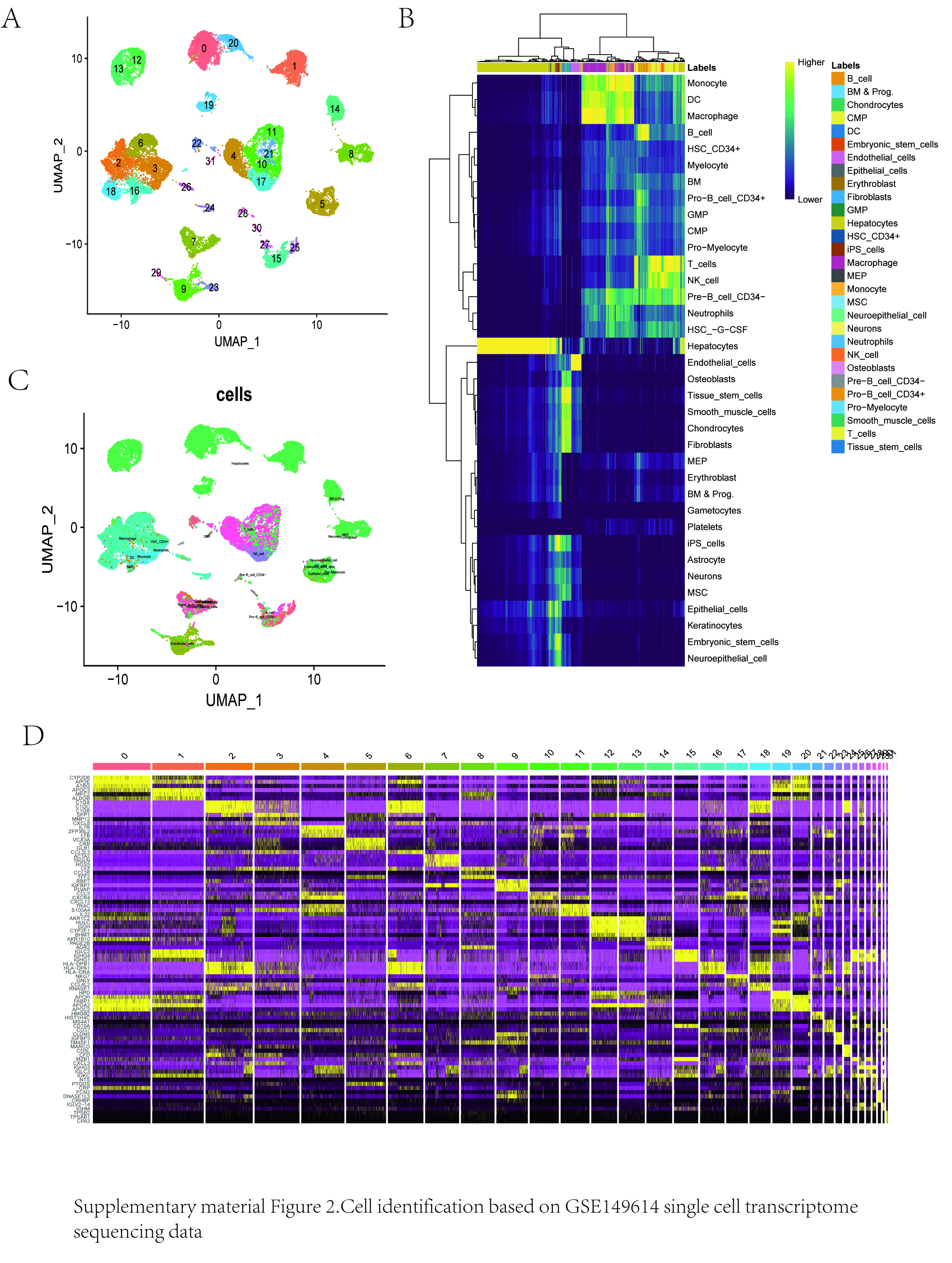

Supplement: Supplementary file 9 [file Image_2.TIF]

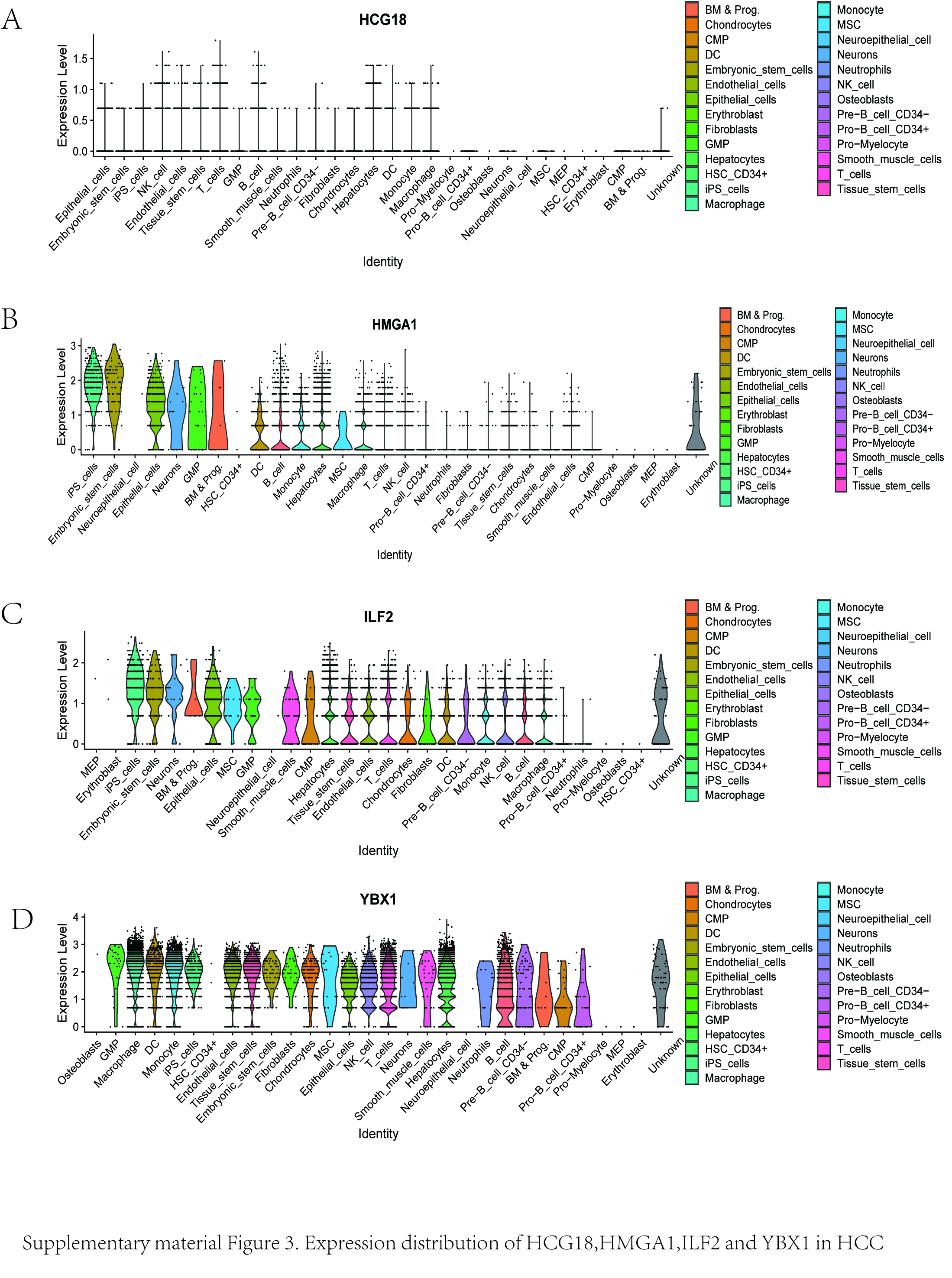

Supplement: Supplementary file 10 [file Image_3.TIF]

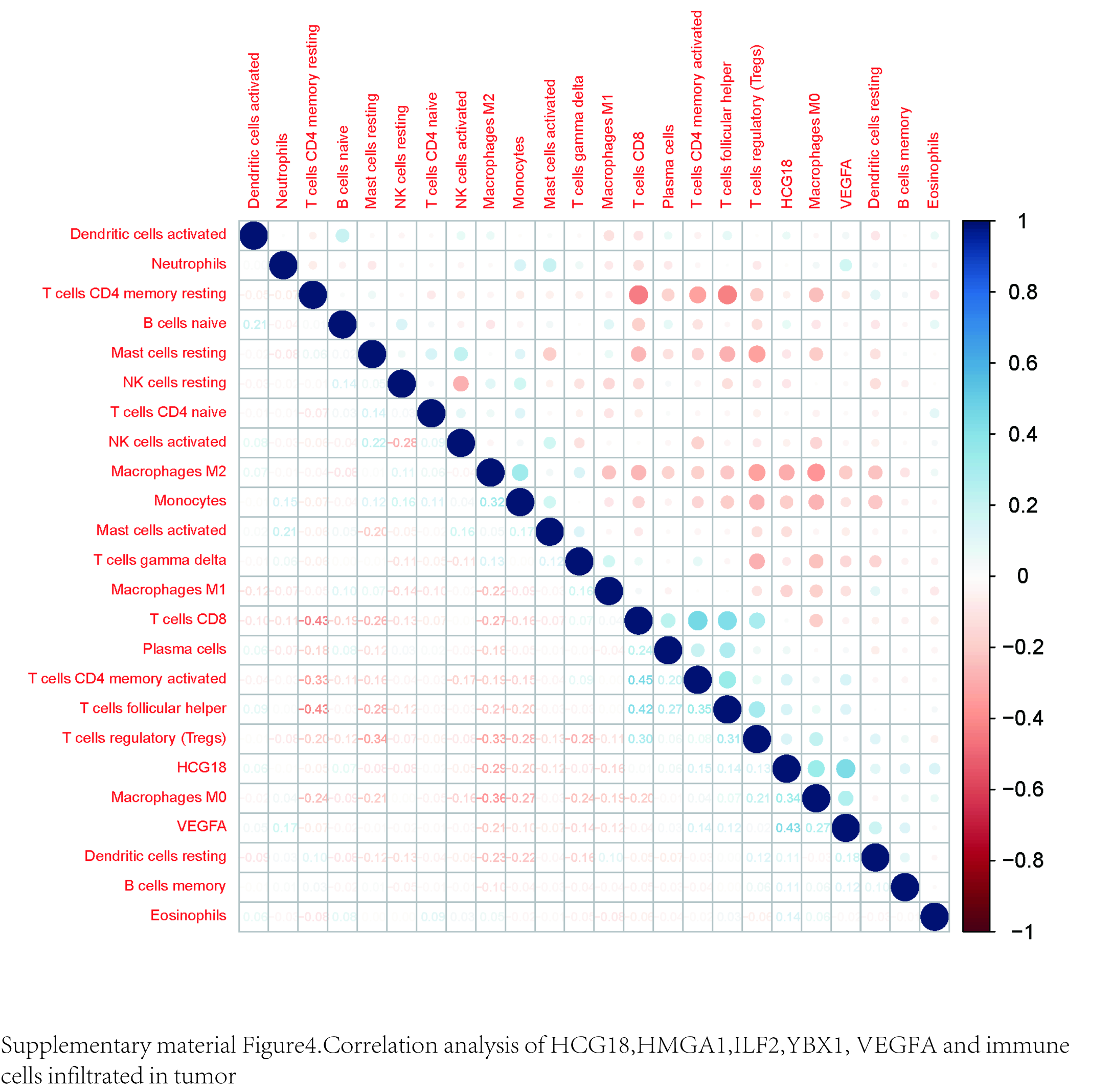

Supplement: Supplementary file 11 [file Image_4.TIF]

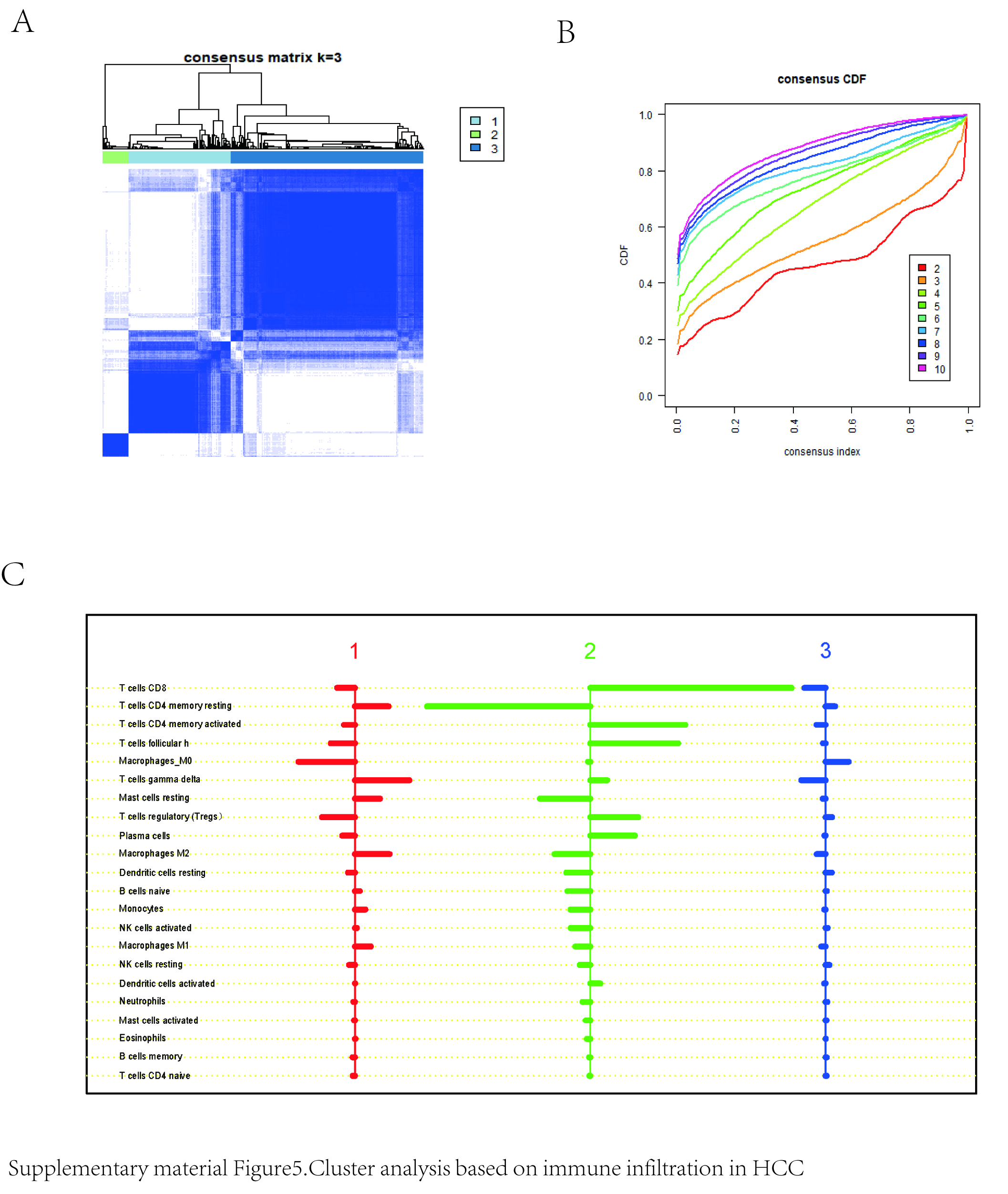

Supplement: Supplementary file 12 [file Image_5.TIF]
